# Evaluation of performance, acceptance, and compliance of an auto-injector in healthy and rheumatoid arthritic subjects measured by a motion capture system

**DOI:** 10.2147/PPA.S160394

**Published:** 2018-04-09

**Authors:** Xiao Xiao, Wei Li, Corbin Clawson, David Karvani, Perceval Sondag, James K Hahn

**Affiliations:** 1 Department of Computer Science, The George Washington University, Washington, DC; 2 MedImmune, LLC, Gaithersburg, MD; 3 Arlenda, Inc., Flemington, NJ; 4 Department of Pediatrics, The George Washington University, Washington, DC, USA

**Keywords:** subcutaneous injection, auto-injection, motion tracking, hand disability, rheumatoid arthritis, Cochin score

## Abstract

**Purpose:**

The study aimed to develop a motion capture system that can track, visualize, and analyze the entire performance of self-injection with the auto-injector.

**Methods:**

Each of nine healthy subjects and 29 rheumatoid arthritic (RA) patients with different degrees of hand disability performed two simulated injections into an injection pad while six degrees of freedom (DOF) motions of the auto-injector and the injection pad were captured. We quantitatively measured the performance of the injection by calculating needle displacement from the motion trajectories. The max, mean, and SD of needle displacement were analyzed. Assessments of device acceptance and usability were evaluated by a survey questionnaire and independent observations of compliance with the device instruction for use (IFU).

**Results:**

A total of 80 simulated injections were performed. Our results showed a similar level of performance among all the subjects with slightly larger, but not statistically significant, needle displacement in the RA group. In particular, no significant effects regarding previous experience in self-injection, grip method, pain in hand, and Cochin score in the RA group were found to have an impact on the mean needle displacement. Moreover, the analysis of needle displacement for different durations of injections indicated that most of the subjects reached their personal maximum displacement in 15 seconds and remained steady or exhibited a small amount of increase from 15 to 60 seconds. Device acceptance was high for most of the questions (ie, >4; >80%) based on a 0–5-point scale or percentage of acceptance. The overall compliance with the device IFU was high for the first injection (96.05%) and reached 98.02% for the second injection.

**Conclusion:**

We demonstrated the feasibility of tracking the motions of injection to measure the performance of simulated self-injection. The comparisons of needle displacement showed that even RA patients with severe hand disability could properly perform self-injection with this auto-injector at a similar level with the healthy subjects. Finally, the observed high device acceptance and compliance with device IFU suggest that the system is convenient and easy to use.

## Introduction

Drug-delivery devices have evolved over the years from traditional syringe systems to advanced patient-operated drug-delivery devices such as auto-injectors. Studies have found that auto-injectors are generally viewed as more convenient than traditional syringes because they provide a number of benefits, including a reduced risk of injection site reactions, reduced discomfort, and greater ease of use.^1^,^2^ Prefilled, disposable auto-injectors have the advantage of simplicity, as they automatically insert the needle and deliver a controlled dose of drug into the skin while requiring minimal training for patients. Despite these advances, patients with reduced manual dexterity (such as rheumatoid arthritic [RA] patients) or other deficiencies may experience complications following correct procedures with these auto-injection devices. Thus, it is desired to analyze more carefully the way users manipulate and use the auto-injector.

Previous research on auto-injectors was primarily focused on the evaluation of the device acceptance and usability. Device acceptance is concerned with the device’s ease of use, safety, and patients’ willingness to adopt the device. The methods to assess the device acceptance were mostly based on survey questionnaires reported by participants^3^–^6^ or collected from physicians or nurses.^7^–^9^ The device usability, which is patients’ compliance with device instruction for use (IFU), was evaluated by independent observers who monitored patients’ handling of the auto-injector system during injections.^10^,^11^

Besides device acceptance and usability, capturing and analyzing the performance of self-injection with auto-injectors is essential in acquiring precise data concerning the procedure and also in quantifying the performance. In Berteau et al’s^12^ study, accuracy and consistency of the injected volume, skin reaction, pain, and fluid depot in the hypodermis layer were quantitatively measured by gravimetric methodology, visual analog scale, and ultrasound sonography, respectively. Since the perceived pain on injection site may cause negative effects on acceptance and adherence to treatment in patients with chronic diseases, auto-injectors with different needle lengths were analyzed to minimize the injection pain.^13^ Moreover, motion capture systems have previously been used in many medical applications for quantitative assessment of surgical skills and dexterity during laryngoscopic, arthroscopic, and open procedures.^14^,^15^ Motions were captured by electromagnetic sensors,^16^ infrared cameras,^17^ or Kinect^®^ sensors.^18^ These methods all have advantages in terms of objectively recording, analyzing, and visualizing motion data. However, to the best of our knowledge, no studies have been performed for evaluating motion capture data in injection-related procedures.

In this study, our objective was to show that motion analysis could contribute to evaluate subjects’ performances of self-injection with auto-injector. The performances of subjects were quantified by calculating the needle displacement during the simulated injection. Comparisons were made between healthy and RA subjects. In addition, device acceptance and usability were also assessed in this study.

## Methods

### Study design and setting

This study was conducted in the research suite at Shugoll Research, Bethesda, MD, USA. The suite consists of an interview room and a viewing room. All injections were performed in the interview room while independent observations were made in the viewing room. A total of 38 subjects were recruited in the study, including nine healthy volunteers and 29 RA patients with different levels of hand dysfunction. The research protocol was approved by an internal committee from the Patient Safety Organization, Quality Organization, Regulatory Organization, and Development Organization at MedImmune and complies with principles expressed in the Declaration of Helsinki. According to the guide that was published by Ximedica, an independent and industry leader in human factors research, we assessed the risk of this study to participants to be low. At this risk level, Institutional Review Board approval was officially waived by MedImmune. Written informed consent was obtained from the participants.

Each subject was asked to perform two simulated subcutaneous self-injection using a foam pad attached to the most common injection sites (one on the thigh, the other on the abdomen) in random order. As an exploratory objective to increase the difficulty of injection, we also randomly selected four healthy subjects to perform an extra injection while wearing gloves specifically designed to limit hand dexterity. Two observers were present in this study and directly observed all activities. In order to standardize the observations, the observers used a written guide as a reference to record the same set of information for all the subjects (Table 1). The interview room simulated the environment that would normally be encountered in the subjects’ homes or other appropriate environments. We also videotaped all performances with an external video camera.

**Figure 1:**
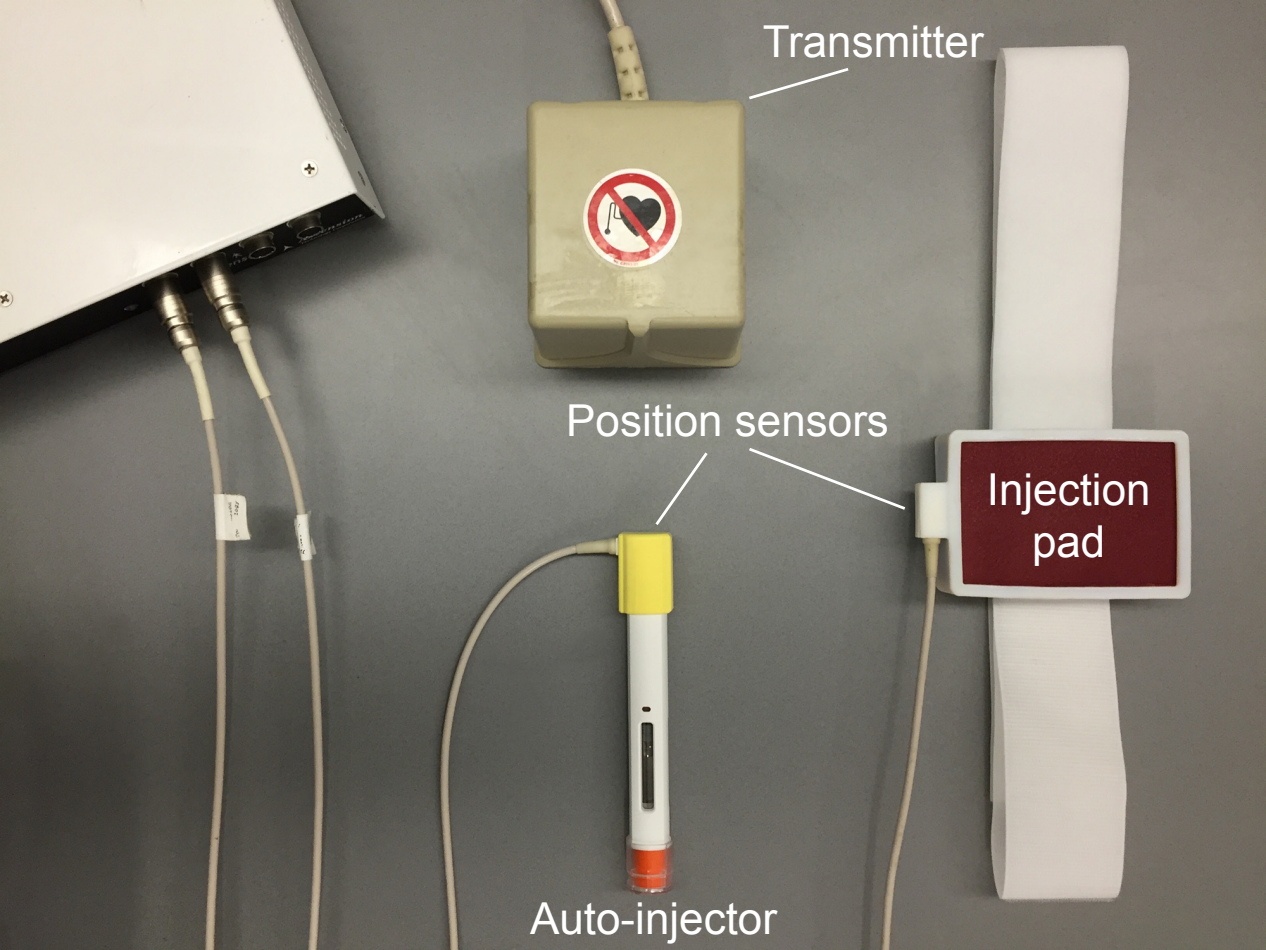
Experimental setup with the injection device.

To capture the performances of injections, we used the 3D Guidance trakSTAR^®^ electromagnetic motion capture system (Ascension Technology Corporation, Shelburne, VT, USA) to continuously track the positions and orientations of the injector and the injection pad (Figure 1). One sensor was attached to the top of the injector (to avoid physical interference with the subject’s hand). Another was attached to the side of the injection pad. The transmitter with an operating range of ~1 m diameter hemisphere was placed in a fixed position and was used as a coordinate reference. We eliminated any magnetic materials in the interview room to assure the accuracy of the position sensors. All the sensor cords were organized in order not to block the view of subjects or interfere with their motions. The auto-injector is a typical prototype of many commercially available devices.

**Figure 2:**
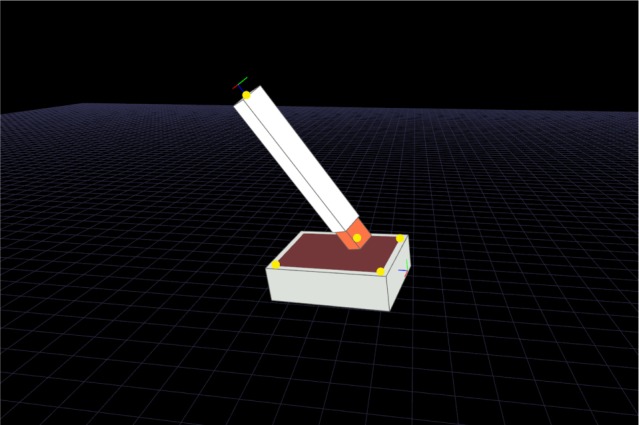
Virtual fiducials used for calibration.

Six degrees of freedom (DOF) motions of the injector and the injection pad were recorded at 60 Hz during injections. A calibration procedure was performed before the experiment by using a 1.5 mm cylindrical sensor from the same motion capture system. We used its tip to measure specific *x*, *y*, and *z* positions on the injector and the injection pad. Two points were selected on the injector, including the top center of the injector and the needle tip of the injector (yellow dots in Figure 2). To determine the motion of the injector with respect to the injection pad, three additional points were selected at each corner of the injection pad. The local coordinate system of the injection pad was defined by these three points, which were sufficient to define an origin and the *x*-, *y*-, and *z*-axes. The *z*-axis was defined as the vector that was perpendicular to the injection pad. The *x*–*y* plane was defined as the pad surface. Given the positions of the two sensors that we attached to the injector and the injection pad, we could further calculate the positions of the five selected points. The motion capture system was connected to a personal computer through a universal series bus (USB) port. Data acquisition, analysis, and 3D visualization of the data were implemented using software developed in our laboratory.

The study started with the RA subjects filling out the Cochin scale,^19^ a practical instrument for rating hand disability in RA patients. It comprised 18 questions on daily activities, each scored from 0 (without difficulty) to 5 (impossible to do). A total score obtained by simply adding the scores of the 18 questions would reflect each patient’s hand function severity, with a higher score representing more severe hand dysfunction. Other information, such as gender, previous self-injection experience, and pain severity in hands (only for RA subjects, scored from 0 [no pain] to 5 [severe pain]), was also reported by all the subjects. The interviewer gave each subject instructions on how to properly use the device before the first injection. All the subjects were asked to hold the injector in place for 60 seconds while seated in a chair even though the time for a full dose is typicallŷ10 seconds. This was done in order to analyze the motion for extended injections. During each injection, the motion data were streamed and exported to the computer for further analysis. An eight-step injection checklist was used to help the observers record subjects’ noncompliance with the injection procedure, including errors when subjects handling the auto-injector and performing the injection (Table 1). After the injection, a survey questionnaire containing 12 questions was used to assess the device acceptance (Table 2). To evaluate the perceived ease and confidence of using the device, subjects completed the first four questions (Q1–Q4) after both injections. At the end of the second injection, eight more questions (Q5–Q8) were administered concerning the overall experience of the injection process and the device design.

### Data analysis

Data were analyzed using software developed in-house, Matlab^®^ (Mathworks, Natick, MA, USA) and JMP^®^ Version 12 (SAS Institute Inc., Cary, NC, USA). The performance of injection, device acceptance, and usability were evaluated based on the motion we collected from the motion capture system and the survey questionnaires correspondingly.

#### Measurement of the injection performance

Human skin has been demonstrated to have elastic properties and is subject to the mechanical laws defining its properties.^20^ In the elastic range, the property is expressed by Young’s modulus: *E*=*σ*/*ε*, where *σ* is defined as stress and *ε* is defined as strain. In addition, *σ*=*F*/*A*, where *F* is the applied force and *A* is the original cross sectional area and *ε*=*Δl*/*l*, where *Δl* is the change in length and *l* is the original length. Therefore, the change in length of the skin (*Δl*/*l*) is proportional to the force (*F*) applied to it. In our study, we used needle displacement as an indication of the amount of force that the subject applied to the injector in the direction of the displacement. Considering that there is no known maximum acceptable force on the injection point and that limit may vary from one subject to another depending on age, anatomic locations, and other factors, this analysis was mainly for measuring the relative forces applied on the skin by the subjects.

**Figure 3:**
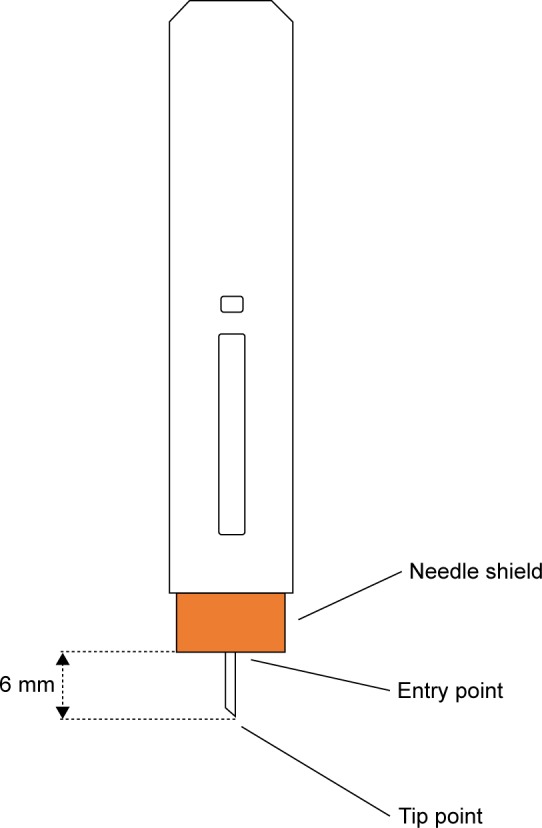
The state of the auto-injector when fully inserted into skin.

In order to calculate the needle displacement, two points were defined on the needle of the injector: tip point and entry point. The tip point was defined as the tip of the needle. The entry point was defined as the intersection of the needle with the pad surface (Figure 3). In this particular device, when injection starts, instead of the needle moving forward, the plastic shield (orange part in Figure 3) retracts and stops when the safety mechanism clicks. The exposed part of the needle is 6 mm, which is the distance from the entry point to the tip point. We make the assumption that movements in the direction parallel to the needle cause very little stretch or compression to the skin since there is very little friction between the needle and the skin. The relevant needle displacement can be measured by the displacements of the entry and tip points.

**Figure 4:**
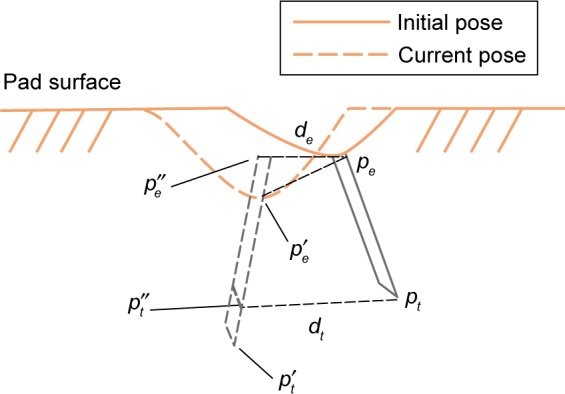
An illustration for calculating the needle displacements of the entry point and the tip point (*d_e_*, *d_t_*) from the initial pose (*p_e_*, *p_t_*) to the adjusted current pose (*p_e_″*, *p_t_″*). (*p_e_′*, *p_t_′*) is the original current pose.

For our study, we analyzed the motion within the duration of the injection, which was defined as the time (in seconds) from when the needle of the injector was fully inserted into the injection pad until the timer rang (60 seconds). We extracted the segment of the duration of injection from the motion trajectory and set the first needle pose as the initial pose (Figure 4). Then, we calculated the displacement of the entry point *d_e_* and the displacement of the tip point *d_t_* with respect to the initial pose for all subsequent needle poses during the 60 seconds’ injection. Figure 4 illustrates the calculation of needle displacement between the initial needle pose and one of the subsequent needle poses. We first transformed the entry/tip points of both the initial needle pose (*p_e_*, *p_t_*) and the current needle pose (*p_e_*′, *p_t_*′) to the local coordinate system of the injection pad to determine the motion of the needle with respect to the injection pad. Then, we moved the current needle pose in the direction of its needle pose to the same level as the pad surface at the initial needle pose. The resulting pose was the adjusted needle pose (*p_e_*″, *p_t_*″). Finally, the displacements of the entry point *d_e_* and the tip point *d_t_* were calculated as the Euclidean distance between the initial and the adjusted needle poses.

By knowing the displacements of the entry point and the tip point for each pose during the injection, we determined a time series trajectories of needle displacements for each injection. Comparisons regarding max, mean, and SD of needle displacement in 60 seconds’ injection were made between the healthy group and the RA group on both thigh and abdomen data. A nonparametric test, Wilcoxon rank sum, was used to analyze the mean difference in each comparison. Subgroup analyses of the mean needle displacement in the RA group were carried out based on gender, previous experience in self-injection, grip method, injection site, pain in hand, and Cochin score. Generalized estimating equation was used to account for the repeated measures. Analyses of needle displacements for different durations of injection were also carried out to determine if the subjects could hold the injector in place for an extended time (ie, 15, 30, 45, and 60 seconds).

#### Evaluation of the device acceptance and usability

The device acceptance was evaluated based on the results of the survey questionnaire. We computed mean score (±SD) for the 5-point Likert scale questions (Q1, Q2, Q5, Q6, Q7, and Q8) and percentage of acceptance for the binary questions (Q5.a, Q6.a, Q7.a, and Q8.a). For question Q3, we counted the percentage of subjects who felt they had not made a mistake. For question Q4, we calculated the percentage of subjects who thought they would be able to perform the injection even when arthritis in their hands was exceptionally painful. A score of ≥4 or a percentage of ≥80 was considered as high acceptance.

The device usability was evaluated by the observers who monitored the handling of the auto-injector and recorded each error that occurred during execution of the injection steps. The IFU compliance was assessed by analyzing the percentage of injections where all steps were correctly performed. An overall mean percentage was calculated for the first injection and the second injection. The mean percentage in the RA group was calculated. A percentage of ≥80 was considered acceptable.

## Results and discussion

All 38 subjects, including nine healthy volunteers and 29 subjects with RA, were analyzed in our study. Among the RA subjects, 19 subjects had experiences with self-injection (using a needle and syringe and/or an auto-injector) prior to inclusion in the study while 10 subjects had no experience. All of the RA subjects had Cochin scores ≤70, of whom 17 subjects had mild hand disability (<30) and 12 subjects had severe hand disability. Motion data were collected for all of the subjects, with the injection procedure being performed twice on a different injection site for each subject. A total of 76 data sets with four additional attempts with dexterity-limiting gloves were analyzed.

### Comparisons of needle displacement

**Figure 5:**
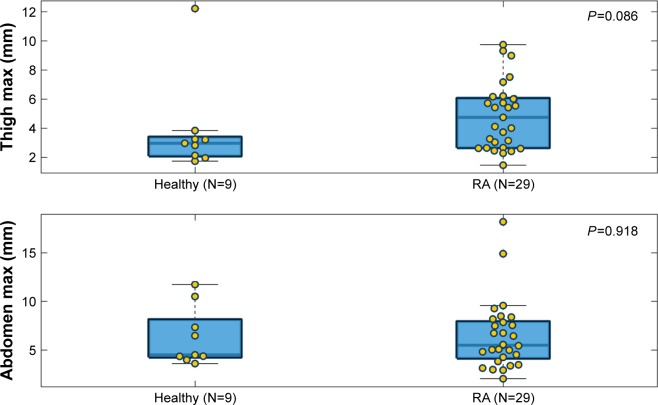
Comparison of max needle displacement between the healthy group and the RA group based on thigh and abdomen measurements. **Abbreviation:** RA, rheumatoid arthritics.

**Figure 6:**
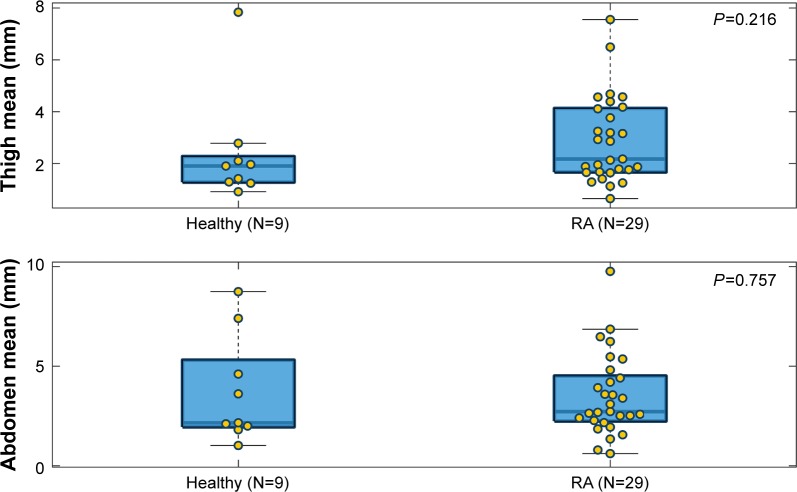
Comparison of mean needle displacement between the healthy group and the RA group based on thigh and abdomen measurements. **Abbreviation:** RA, rheumatoid arthritics.

**Figure 7:**
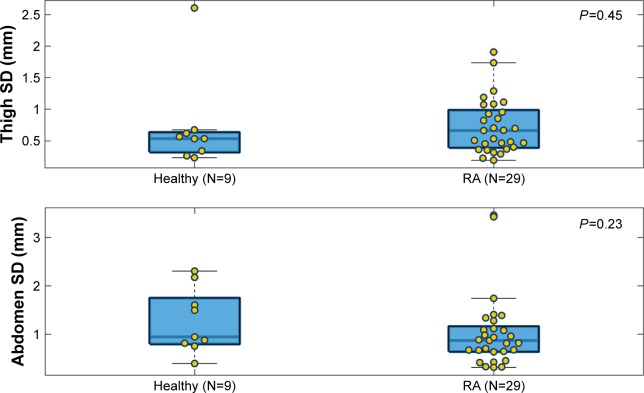
Comparison of SD of needle displacement between the healthy group and the RA group based on thigh and abdomen measurements. **Abbreviation:** RA, rheumatoid arthritics.

The max, mean, and SD of needle displacement of each injection attempt were plotted separately (Figures 5–7). In each boxplot, we compared the needle displacement between the healthy group and the RA group based on thigh and abdomen measurements. The actual data values are shown as yellow dots, spreading out laterally to avoid overlap. The body of the box extends from the 25th percentile to the 75th percentile of the data (so half the points fall within the box) with a thick line marking the median. The whiskers that extend on either side of the box represent a region that should contain most of the points. In our analysis, the maximum whisker length was set to 1.5. Points beyond the whiskers are considered outliers (±2.7 SD).

For the max and mean needle displacement, clear differences were found on both thigh and abdomen measurements, with higher medians in the RA group. This indicated that subjects in the RA group had a higher needle deflection than the healthy group. However, the differences did not reach statistical significance (*P*=0.074 for thigh max and *P*=0.918 for abdomen max; *P*=0.257 for thigh mean and *P*=0.81 for abdomen mean). A significant difference was not found in the SD between the healthy group and the RA group (*P*=0.45 for thigh SD and *P*=0.257 for abdomen SD). Across all the comparisons, we notice that the range of values is larger for abdomen measurement than for thigh measurement, which indicates that the subjects had more difficulty keeping the injector angled correct (as reflected in the max/mean of needle displacement) and steady (as reflected in the SD of needle displacement) on the abdomen.

**Figure 8:**
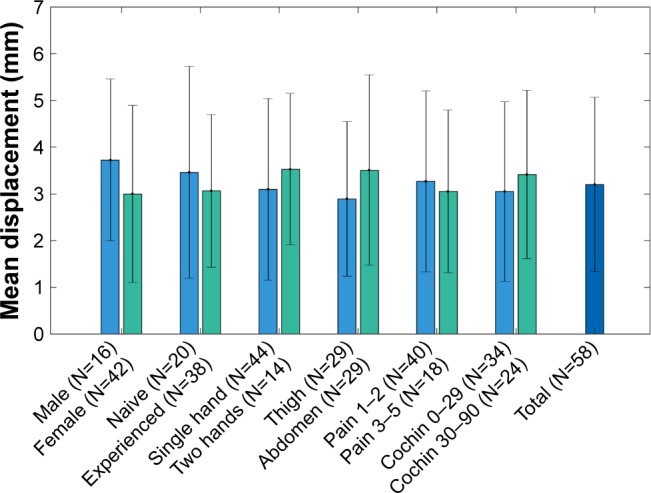
Comparison of mean needle displacements between subgroups in RA subjects. **Abbreviation:** RA, rheumatoid arthritics.

The subgroup analyses of the average needle displacement based on gender, previous experience in self-injection, grip method, injection site, pain in hand, and Cochin score were performed (Figure 8). The results showed significant differences between the subgroups regarding gender and injection site. Specifically, the male group had larger needle displacement than the female group (*P*=0.002). However, this effect was not clinically significant. Injections performed on abdomen had larger needle displacement than the injections performed on thigh (*P*=0.025). This result was in accord with previous comparisons on the needle displacement between the healthy group and the RA group, which shows that it is more challenging for subjects to perform injections on the abdomen than on the thigh. There were no statistically significant differences between the rests of the subgroups (*P*>0.05). In particular, the Cochin score had no significant impact on the performance of injection (*P*=0.249), which means that the degree of hand disability is not an indication of deteriorated self-injection capability with the auto-injector.

**Figure 9:**
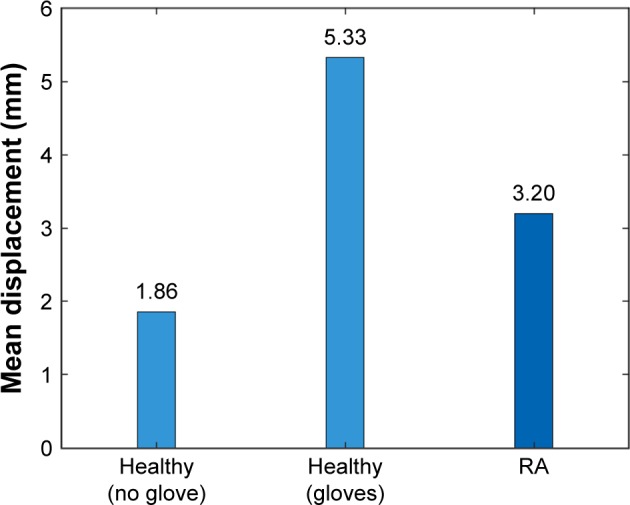
Comparison of mean needle displacement with or without gloves. **Abbreviation:** RA, rheumatoid arthritics.

Figure 9 illustrates the comparison of mean needle displacements between healthy volunteers who performed extra injections by wearing dexterity-limiting gloves and RA subjects. We also included the average needle displacement of these healthy volunteers’ previous two injections as a reference. We can conclude that injection with gloves did limit hand dexterity and lead to deteriorated injection performance (larger needle displacement).

**Figure 10:**
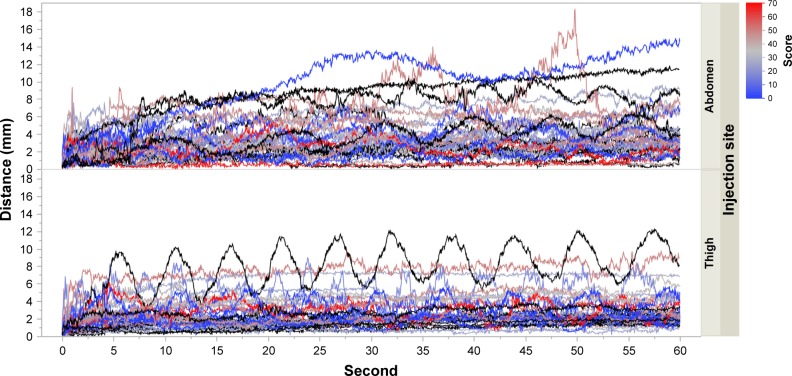
Needle displacement at every 1/60 second for each subject, split by injection site. **Notes:** Black curves represent healthy subjects, and colored curves represent RA subjects. The color scale (blue to red) indicates the severity of the disease (low to high). **Abbreviation:** RA, rheumatoid arthritics.

**Figure 11:**
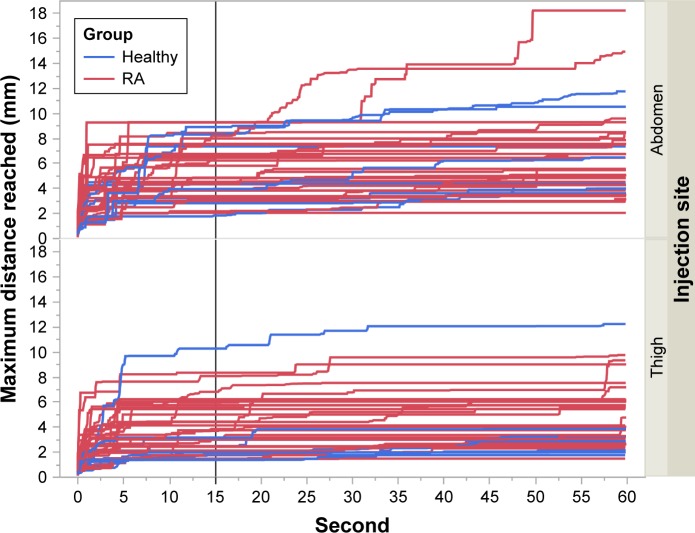
Maximum distance reached up to time *t*. **Notes:** The vertical line is *t*=15 seconds. Blue curves represent healthy subjects, and red curves represent RA subjects. **Abbreviation:** RA, rheumatoid arthritics.

The needle displacements for different durations of injection were also analyzed to determine the performance for longer injections. As there is no known maximum acceptable displacement and that limit may vary from one subject to another, this analysis was used to compare the performance of different populations. The needle displacement at every 1/60 seconds for each subject is presented in Figure 10. We observed that there was no major difference between the healthy and the RA subjects. We then analyzed both groups together. Considering the complexity of the data, we did not fit a statistical model. Therefore, the analysis was done on the maximum displacement reached at every time point (Figure 11).

We calculated the proportion of subjects that reached their maximum displacement at 15 seconds, as well as the proportion of subjects that reached the 50th, 75th, 90th, and 95th percentile of their displacement at 15 seconds (Table 3).

**Figure 12:**
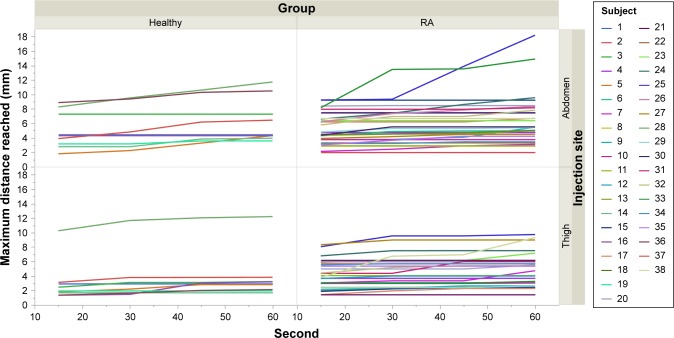
Maximum distance reached at 15, 30, 45, and 60 seconds per subject. **Note:** Healthy subjects are on the left and RA subjects on the right. **Abbreviation:** RA, rheumatoid arthritics.

The results showed that ~60% of the subjects reached the 90th percentile of their personal displacement within the first 15 seconds. To determine if the displacement would significantly increase >15 seconds, we observed the maximum displacement reached within the first 15, 30, 45, and 60 seconds of the injection (Figure 12). The results indicated that most subjects’ displacement at 60 seconds was almost the same as the displacement that they reached at 15 seconds. Except in some rare cases, the increase of the displacement after 15 seconds was quite small.

The above analysis shows that increasing the duration of the injection above 15 seconds did not significantly affect the maximum needle displacement. However, caution needs to be applied in the interpretation of the results. Because of the complexity of the data, no statistical inference had been performed. Also, since the subjects injected the needle in the pad, pain was not a factor.

### Device acceptance and usability

The mean scores (±SD)/percentages of all the survey questions were calculated for both all the subjects and the subjects in the RA group (Tables 4 and 5). Device acceptance was high for most of the questions (ie, >4; >80%) except Q3 and Q5 (ie, >3; >68%). Q3 was related to the probability of the subjects feeling like they would make a mistake. As reported by the subjects, some felt like they made a mistake because they could not decide whether the correct dose was given or whether a dose had been given at all. This was because they failed to monitor the viewing window on the injector, which indicated the volume of the dose. However, the percentage for Q3 increased after second injection, which indicated that fewer subjects made mistakes compared to the first injection. Q5 was about the difficulty of removing the cap of the injector. Most subjects complained that it required a lot of force to remove the cap, especially those RA subjects who had severe hand disability. However, when they were asked whether this amount of force to remove the cap was acceptable or not (Q5.a), a high acceptance (86.84% for overall, 82.76% for RA subjects) was achieved because they thought the amount of force to open the cap is necessary due to safety considerations.

Device usability was also evaluated based on the percentage of injections where all steps were correctly performed for both the first injection and the second injection. The mean percentage of correctly executed steps for the overall subjects was 96.05% for the first injection and 98.02% for the second injection. In the RA group, the corresponding mean percentages were 95.69 and 98.71. We can find that after second injection, a higher usability was achieved than the first injection both for all the subjects and the RA subjects, which may have been due to greater familiarity with the device. According to the notes made by the observers, the step that the subjects failed most frequently was placing the viewing window in their line of sight (S3 in Table 1).

## Conclusion

In this study, we developed the first, to the best of our knowledge, motion analysis system to objectively measure simulated self-injection with an auto-injector. We demonstrated the feasibility of tracking the motions of injection to compare the performances between healthy and RA subjects. The quantitative analysis of needle displacement showed a similar level of performance among all the subjects with slightly larger, but not statistically significant, needle displacement in the RA group. Subgroup analyses showed that previous experience in self-injection, grip method, pain in hand, and Cochin score did not have significant effects on the performance of injection. The analysis of needle displacement in different durations of injection showed that most subjects could hold the injector in place without significant increase of displacement from 15 to 60 seconds. However, caution needs to be applied in interpreting the results because the injection was performed in an injection pad and not actual skin. Finally, the observed high device acceptance and percentage of successfully handling the auto-injector (in compliance with the IFU) suggest that the system is convenient and easy to use. However, one limitation of this study was the small sample size, especially the size of the healthy group, which may reduce the stability of the factor analysis and limit the interpretation of the results. The small sample size and the observational nature of the statistical analysis mean that the *P*-values reported in this study should be used to guide interpretation rather than being definitive answers.

## Figures and Tables

**Table 1:** Observation steps

Category	Steps	Injection(s)
Injection procedure	S1. Grasp device relative to injection site with one hand S2. Use the other hand to remove cap S3. Make sure that the viewing window is in your line of sight S4. Position the device at a 90° angle against the skin of the injection site S5. Push auto-injector against the injection site until start click occurs S6. Monitor injection progress and wait for end of injection S7. Hold the device in place for at least 60 seconds S8. Remove auto-injector from injection site in a perpendicular position	Injections 1 and 2

**Table 2:** Survey questionnaire

Category	Questions	Responses	Injection(s)
Injection evaluation	Q1. On a scale of 1–5, where 1 is not at all confident and 5 is very confident, please rate how confident you are that you administered the injection successfully?	Scale from 1 (not at all confident) to 5 (very confident)	Injections 1 and 2
	Q2. On a scale of 1–5, where 1 is very difficult and 5 is very easy, how would you rate the ease of performing an injection?	Scale from 1 (very difficult) to 5 (very easy)	
	Q3. Was there anything about the injection process that caused you to be concerned or to hesitate or that made you feel like you were just about to make a mistake?	Yes/no	
	Q4. Imagine a time in the past when the arthritis in your hands has been exceptionally painful. Do you think you would have been able to perform the injection if you had been feeling that way today?	Yes/no	Injections 1 and 2 (RA only)
Auto-injector experience	Q5. On a scale of 1–5, where 1 is very difficult and 5 is very easy, how easy was it to remove the cap of the auto-injector?	Scale from 1 (very difficult) to 5 (very easy)	Injection 2
	Q5.a. In your opinion, was this amount of ease to remove the cap acceptable or unacceptable?	Acceptable/unacceptable	
	Q6. On a scale of 1–5, where 1 is very difficult and 5 is very easy, how easy was it to press down on the auto-injector?	Scale from 1 (very difficult) to 5 (very easy)	
	Q6.a. In your opinion, was this amount of ease to press down on the auto-injector in place acceptable or unacceptable?	Acceptable/unacceptable	
	Q7. On a scare of 1–5, where 1 is very difficult and 5 is very easy, how easy was it to hold the auto-injector in place for the duration of the injection?	Scale from 1 (very difficult) to 5 (very easy)	
	Q7.a. In your opinion, was this amount of ease to hold the auto-injector in place acceptable or unacceptable?	Acceptable/unacceptable	
	Q8. On a scale of 1–5, where 1 is very difficult and 5 is very easy, how easy was it to grip the auto-injector?	Scale from 1 (very difficult) to 5 (very easy)	
	Q8.a. In your opinion, was this amount of ease to grip the auto-injector acceptable or unacceptable?	Acceptable/unacceptable	

**Abbreviation:** RA, rheumatoid arthritics.

**Table 3:** Percentage of subjects who reached a given percentile of their distance at 15 seconds

Injection site	50th percentile	75th percentile	90th percentile	95th percentile	100th percentile (maximum)
Abdomen	89.19%	78.38%	59.50%	56.76%	32.43%
Thigh	83.78%	67.57%	62.16%	56.76%	29.73%

**Table 4:** Overall device acceptance

Questions	Mean (±SD)/percentage	
	Injection 1	Injection 2
Q1	4.53 (±0.92)	4.76 (±0.59)
Q2	4.70 (±0.56)	4.68 (±0.62)
Q3	73.68%	81.58%
Q4	RA only	
Q5		3.84 (±1.05)
Q5.a		86.84%
Q6		4.76 (±0.49)
Q6.a		97.37%
Q7		4.29 (±0.96)
Q7.a		89.47%
Q8		4.59 (±0.77)
Q8.a		94.74%

**Abbreviation:** RA, rheumatoid arthritics.

**Table 5:** Device acceptance in RA group

Questions	Mean (±SD)/percentage	
	Injection 1	Injection 2
Q1	4.48 (±0.91)	4.72 (±0.65)
Q2	4.62 (±0.62)	4.62 (±0.68)
Q3	68.97%	79.31%
Q4	82.75%	89.66%
Q5		3.90 (±1.05)
Q5.a		82.76%
Q6		4.76 (±0.51)
Q6.a		96.55%
Q7		4.21 (±1.02)
Q7.a		86.21%
Q8		4.50 (±0.85)
Q8.a		93.10%

**Abbreviation:** RA, rheumatoid arthritics.
